# Estimating health state utilities in hemophagocytic lymphohistiocytosis

**DOI:** 10.1186/s41687-020-00276-9

**Published:** 2021-01-20

**Authors:** Beenish Nafees, Andrew Lloyd, Sarah Dewilde

**Affiliations:** 1 Acaster Lloyd Consulting Ltd, London, UK; 2 Services in Health Economics, Brussels, Belgium

**Keywords:** Hemophagocytic lymphohistiocytosis, Ultra-orphan drugs, Health state utilities, Quality of life, Cost-effectiveness analysis

## Abstract

**Background:**

Hemophagocyti.c lymphohistiocytosis (HLH) is a rare and severe disorder characterized by abnormal activation of the immune system. Primary HLH causes prolonged fever, spleen and liver enlargement, and organ dysfunction, usually in infancy and early childhood and is fatal if left untreated. As effective treatment options emerge, such as emapalumab-lzsg, Health Technology Assessment bodies around the world will assess them in terms of cost-effectiveness. This study was designed to estimate quality of life weights (utilities) for such analyses.

**Methods:**

Vignettes were developed describing HLH treatment related health states. Health states included active HLH, HLH plus neurological symptoms, receiving chemotherapy, undergoing stem cell transplant (SCT), graft versus host disease (GVHD), cure and end of life care. The vignettes were based on information from in depth interviews with clinical specialists; and qualitative research with four parents of children with primary HLH aged between 1 and 18 years old. The vignettes were then assessed in time trade off (TTO) interviews with members of the UK general public in one on one face to face interviews with trained, experienced interviewers. Preference data were analysed using the generalised estimating equations framework.

**Results:**

Detailed qualitative data captured the substantial burden of this disease for young children. One hundred participants completed the TTO interviews. The utility score for Active HLH was estimated as 0.32 (95% CI, 0.24 to 0.39). Values for other states were HLH plus neurological symptoms (0.27, 95%CI 0.18–0.35), receiving chemotherapy (0.26, 95%CI 0.17–0.34), undergoing SCT (0.18, 95%CI 0.07–0.28), GVHD (0.07, 95%CI -0.04-0.17), cure (0.72, 95%CI 0.67–0.77) and end of life care (− 0.17, 95%CI -0.27- -0.07).

**Conclusions:**

This study has estimated utility weights for seven different HLH related states which are based on detailed input from carers and physicians and have good face validity. There are few other options for collecting these data in an ultra-rare setting.

## Introduction

Hemophagocytic lymphohistiocytosis (HLH) is a rare and severe disorder characterized by inherited mutation and abnormal activation of the immune system [1]. Primary HLH is a genetic disorder which causes prolonged fever, enlargement of the spleen and liver, and organ dysfunction [1–3]. Primary HLH appears usually in infancy and early childhood and is fatal if left untreated [4]. It affects approximately 1 in 50,000 live births in the world per year [1]. Treatment of primary HLH is individualized, and potentially curative for patients. Treatment can start very soon after diagnosis. Active HLH involves immunosuppressive induction therapy, followed by a hematopoietic stem-cell transplant (HSCT), [5]. Induction therapy is associated with significant toxicity, and HSCT can lead to graft versus host disease. The management of the disease and the preparation for HSCT will lead to multiple hospital admissions for patients [5]. The conventional treatment for primary HLH includes 8 weeks of etoposide (chemotherapy) and dexamethasone (coriticosteroids). Patients and their families experience a very significant burden related to the disease, treatments, adverse events and risks, all of which will affect their health-related quality of life (HRQL). Adverse events such as low blood count, nausea, vomiting, and headaches can severely affect patient’s HRQL. More recently a new treatment has emerged for patients called emapalumab-lzsg (Gamifant®) which has been shown to be effective in these patients, especially children and adults with refractory, recurrent or progressive disease. Progressive disease, if left untreated, can lead to failure of multiple organs and patients only survive for a few months after this.

New treatments such as emapalumab-lzsg are reviewed by decision makers in terms of the health gain or value they represent to the health service. Such assessments are driven in part by an economic evaluation, which will examine the cost of the treatment against the gain in health (expressed in terms of years of life and quality of life). Emapalumab-lzsg is a highly specialized treatment which is very high cost but potentially also very high value because of its potentially curative effect. Decision making by Health Technology Assessment (HTA) bodies is extremely complex for such treatments because the outcomes data are uncertain and there is a high opportunity cost [6]. HTA bodies typically examine the effectiveness, and cost-effectiveness, of a health technology such as a drug or a medical device.

This study is designed to estimate utilities for primary HLH to help support decision making for all treatments for HLH.

## Methods

### Study design

The study was divided into two parts: 1) health states were developed in the first part of the study involving qualitative, semi-structured interviews with parents of children with primary HLH and clinicians; 2) a time trade-off methodology was used to elicit utility values for each of the health states from a broadly representative sample of the general public in the UK.

### Ethics review

The study protocol was reviewed and approved by an independent review board: Salus IRB (date of approval: 14th August 2018).

### Part 1: health state development

Health state descriptions or ‘vignettes’ were developed through a process that combined information gathered from consultation with clinical specialists and in-depth qualitative interviews with parents of children with primary HLH aged between 1 and 18 years old. This is a standardized approach which has been used in similar studies previously [7, 8]. Qualitative feedback obtained from interviews was used to describe patient’s experience of different states of disease and treatment as per each health state.

### In depth interviews

Parents were recruited through patient support groups and social media such as Facebook pages in the United States (US), which were selected based on previous research in this area and study team. An advertisement was placed on the social media pages and support group newslettet to contact the team by email if potential parents were interested. Participants who contacted the team were then asked to schedule a convenient time to screen. If participants were eligible, they were then asked to schedule a convenient time for a telephone interview. Three parents were recruited from Facebook support page created by parents and one parent was recruited from a support group. Potential participants included people who reported that they were a parent or primary caregiver of a child (< 18 years) who had been diagnosed with HLH that required treatment with a stem cell or bone marrow transplant within the previous 10 years. All parents (*N* = 4) were from the US and all interviews were conducted in English. The parents talked about the hospital where their child received care and provided details that were consistent with their child having HLH. One round of interviews was conducted with parents and two rounds of interviews were conducted with clinicians. Clinicians (*N* = 3) were recruited who had experience of managing and treating patients with primary HLH. The clinicians were all known to the study sponsor. All participants provided written consent and then took part in a semi-structured, telephone interview. The parent interviews explored the circumstances which led to paitent’s diagnosis, patient’s experience of HLH symptoms, the treatment and burden that the disease has on his/her quality of life. The interviews lasted approximately an hour. Parents were also asked to describe the treatment and complications that their child had experienced.

The first round of interviews explored the impact of primary HLH and the types of treatment available and associated adverse events. Physicians discussed management of HLH and known adverse events. Based on the first round of interviews, draft health state descriptions were developed. These were validated in the second round of interviews with clinicians for accuracy. All Interviews were recorded and transcribed. Parents received a $50 Amazon voucher for their participation.

The information was summarized by one team member and reviewed additionally by a second team member, without any use of software. Key information from interviews with parents and clinical experts was compiled and summarised in terms of key areas of functioning (usual activities, emotional wellbeing, social functioning, cognitive ability), symptoms and quality of life. If symptoms, areas of functioning and aspects of quality of life were mentioned by more than one participant, they were included in the draft health states. For each state the intention was to capture sufficient information to be able to describe the symptom burden (pain, fatigue etc), the psychosocial impact and the impact on physical functioning. Interviews were summarised in terms of these different aspects of HRQL. Information was collected and included if it was reported by more than two parents and by at least one clinician. From these interviews, the draft vignettes were derived which represented a typical life cycle of treatment. These included active HLH, active HLH receiving chemotherapy, active HLH with neurological (CNS) involvement in the form of seizures, undergoing stem cell transplant, successful treatment or cure, graft versus host disease, and receiving end of life care.

### Expert review

Two further expert interviews were conducted to review the content of the vignettes. Clinicians were asked to comment on how appropriate anad accurate each description was. Changes to the wording of the vignettes were suggested to improve the accuracy of the health states. These changes were incorporated and the final vignettes used for the valuation exercise are presented in the Appendix.

### Part 2: health state vignette valuation

Members of the UK general public were recruited to take part using newspaper advertisements and an existing database of volunteers. The general public were recruited to provide a societal perspective on the impact of HLH. Societal perspectives are generally preferred by HTA bodies because they are making recommendations regarding the public’s access to health care. The sample was designed to reflect the general population in terms of age and gender. All interviews were conducted by trained interviewers. All participants gave written informed consent. Participants completed a brief socio-demographic questionnaire and the Time trade-off (TTO) interview. The TTO interview assesses the value or worth of different states of health by exploring how many years of life people may be willing to sacrifice in order to avoid such a state. It is generally preferred over other approaches (e.g. standard gamble) [6]. The TTO method is a preferred approach by assessment bodies such as NICE [6].

During the TTO exercise, all participants first rated the vignettes on a scale of 0 (worst imaginable health) to 100 (full health) on a visual analogue scale (VAS). Participants were asked to read the vignettes one at a time, including a state called ‘dead’ and place them on this scale. In the TTO task participants imagined that they were currently experiencing each health state (described in the vignette) and they were asked to choose whether they preferred: (1) to live in the health state for a period of 10 years followed by death; (2) to live for X number of years in full health; or (3) to indicate that the two previous options were equally desirable. Time in the state of full health (X) was systematically reduced from 10 downwards until the respondent was indifferent between the two choices.

### Analysis

Descriptive statistics were used to present socio-demographic data such as age, gender, qualification and employment. The VAS and TTO data were analysed separately using regression modelling using SAS software version 9.4. In these analyses, the dependent variable was a transformation of the VAT and TTO values, to transpose left-skewed utility data into right skewed dependent variables so that distributions could be more easily fitted to the data. These transformations consisted in changing each value into its complement: *TTO complement = (1- TTO utility) and VAS complement = (100- VAS value)*. In order to obtain the actual utility values for each health state, the opposite linear transformation needs to be applied: *TTO utility = 1 - TTO complement, and VAS value = 100 - VAS complement.*

The independent variable in each regression was the health state, in order to obtain a utility value with a 95% confidence interval for each HLH-related health state. Based on these regressions, the mean TTO and VAS values (and 95% confidence intervals) were generated for each health state. In a second phase, gender, age, employment and education were also included in the regression, to understand whether any of the respondent characteristics had an influence on the results.

The analysis was carried out in the General Estimating Equations framework, which is suitable for analysing correlated data. As each respondent evaluated the full set of health states, these evaluations are correlated within the subject. Several forms of the correlation matrix between the repeated measurements were fitted: independent, exchangeable, compound symmetry and unstructured correlation matrix. Furthermore, models were estimated with an identity or a log link, and with a normal or Gamma distribution for the error terms. These models were tested against each other using the quasi-likelihood under the independence model criterion (Quasi Information Criterion or QIC) which was developed by Pan for model selection in a GEE environment [9, 10]. This QIC statistic works in an analogous way to Akaike’s information criterion (AIC) in that the best fitting model is that with the lowest value of QIC after paying a penalty for the number of parameters fitted [11].

## Results

### Qualitative results

Four caregivers in the US and 3 clinicians (US and Europe) were interviewed. The clinicians had been treating children with HLH for at least 10 years and were seeing between 3 and 10 patients with primary HLH a year which is a high number for primary HLH. In the four parent interviews, discussion focused on their children (now aged between 3 and 12 years) who had all been diagnosed with primary HLH at least over a year ago and were in remission or fully recovered. Three of four children were diagnosed within the first year of birth, and one child was diagnosed at the age of 3 years. All children were now in remission. All parents described that their children presented a prolonged fever that lasted several days and fatigue, before they went to hospital. At hospital, diagnosis was made after tests were conducted. One parent reported that there was a 6 month delay in the hospital understanding that she had HLH.

Clinicians described the presentation of a ‘typical’ patient with primary HLH (high, persistent fever, skin rash, low energy, generally feeling unwell and enlarged spleen or liver). The clinicians described the standard treatment regimen as ‘toxic and aggressive’. They described how children in the US were usually hospitalised for 6–8 weeks after which they will continue to receive treatment as an outpatient if they are improving. With a survival rate of 40%, patients who survive the usual treatment phase would be eligible to receive a stem-cell transplant which could lead to one of three outcomes; a successful cure, rejection of graft leading to second transplant, or rejection of graft leading to death.

Treatment required children to be in isolation due to the risk of infection. Parents reported that young children felt distressed, frustrated and angry as they couldn’t play or go outside, and they were ‘hooked onto IV treatment’. Parents reported how the children are unable to do usual activities that children do. Even when children leave the hospital, they are told to avoid crowded places due to risk of infection, for up to one year.

An unsuccessful transplant leads to more anxiety, distress and upset. Clinicians reported that patients can have complications from the transplant which can lead to problems with eating or drinking, weight loss, skin rashes, or chronic lung disease. If children have a successful transplant, they can resume some normal activity, e.g. playing, and be mobile, after 3 months and return to hospital for follow up treatments. Their normal nutrition should return after 6 months.

Clinicians reported that approximately 20% of children experience central nervous system involvement. This can be mild and include seizures and loss of balance, moderate involvement such as suffering from epilepsy, or severe such as cerebral palsy. Clinicians reported that the care of patients with neurological problems alongside HLH can be more complicated with more follow-ups, medication, tests and monitoring.

### Valuation results

In the UK, 100 members of general public were recruited. Table 1 presents the demographics for the sample overall. The majority of the sample were female (56%) and between 30 and 40 years of age.

**Table 1:** Socio-demographic profile of the general public sample

Participant characteristics		Current sample ***N*** = 100 (N and %)
Gender Male n (%)		44
Age	< 20 years old	1
	20–30 years old	19
	30–40 years old	42
	40–50 years old	13
	50–60 years old	7
	60–70 years old	15
	70–80 years old	3
Education	None	5
	A levels/ leaving school at 18	14
	GCSE^a^/ leaving school at 16	5
	Graduate	20
	Vocational	8
	University	47
	Not known	1
Main activity	Full time	38
	Self-employed	21
	Part time	13
	Student	6
	Retired	6
	Stay at home	7
	Sick leave	6
	Seeking work	1
	Other	2

**Fig. 1:**
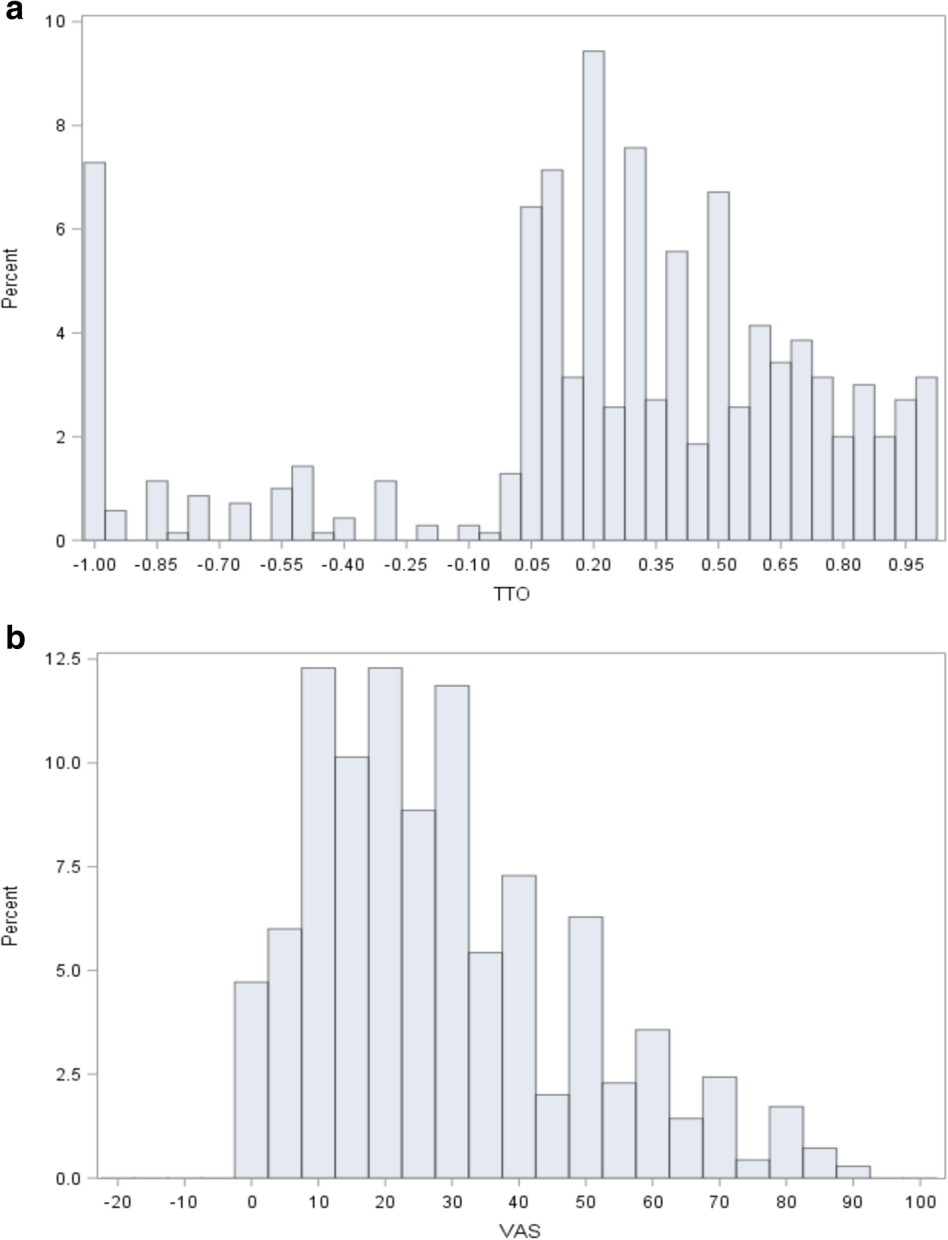
Distribution of the TTO utility weights (**a**), the VAS (**b**). **a**. TTO utility weights. **b**. VAS Values

The distribution of the TTO utilities and VAS values is depicted in Fig. 1**.** The statistical model for the HLH data used the TTO and VAS complements as dependent variables, applied a log link between the dependent variable and the explanatory variables (for the TTO utility weights) or an identity link (for the VAS), and assumed a normal distribution. The model with an exchangeable correlation structure was selected as the most appropriate intra-patient correlation structure. The models tested resulted in similar utility values, which contributed to the robustness of the results. In order to obtain predicted values based on the parameter estimates, the following transformations need to be applied:

$$ {\displaystyle \begin{array}{l} TTO\  utility=1-\mathit{\exp}\ \left( parameter\_ estimate\right).\\ {} VAS\  value=100- parameter\_ estimate.\end{array}} $$

The regression results show that the parameter estimates of all the health states in the model were found to be statistically significant apart from GVHD. Moreover, most utility evaluations of the health states were different from the active HLH health state, as evidenced by the statistically significant contrast statements, except for the chemotherapy health state and the health state with CNS involvement. Based on the two statistical models (Table 2) it was found that the TTO utility for the health state of active HLH was 0.32 (with 95% CI: 0.24 to 0.39) and 31 (95% CI, 27 to 34) for the VAS. This is based on the following calculations with the regression parameters: TTO utility weights for HLH = 1-exp(− 0.3883) = 0.32 and VAS: 100–69.4 = 30.6 (=31 rounded). Other values calculated in a similar way are reported in Table 3.

**Table 2:** Parameters of the regression for the TTO utility weights and for the VAS preference weights for the HLH states

**TTO model for utility decrements**	**Parameter Estimate**	**Standard Error**	**95% Confidence Limits**		**Z**	**Pr > |Z|**
Active HLH	−0.39	0.06	−0.50	− 0.28	−6.96	<.0001
Cure	−1.28	0.09	−1.46	− 1.10	− 13.78	<.0001
End of life	0.16	0.044	0.07	0.24	3.50	0.0005
GVHD	−0.07	0.06	− 0.19	0.04	−1.27	0.2050
HLH + CNS	−0.32	0.06	−0.43	− 0.20	−5.42	<.0001
HLH + chemo	− 0.30	0.06	− 0.42	− 0.19	− 5.08	<.0001
HSCT	− 0.20	0.06	− 0.32	− 0.08	− 3.18	0.0015
**VAS model for preference weights**	**Parameter Estimate**	**Standard Error**	**95% Confidence Limits**		**Z**	**Pr > |Z|**
Active HLH	69.44	1.59	66.32	72.55	43.67	<.0001
Cure	44.94	1.81	41.39	48.50	24.77	<.0001
End of life	85.15	1.19	82.82	87.48	71.55	<.0001
GVHD	79.42	1.45	76.58	82.26	54.85	<.0001
HLH + CNS	72.73	1.67	69.46	75.99	43.65	<.0001
HLH + chemo	71.51	1.68	68.22	74.81	42.57	<.0001
HSCT	76.83	1.55	73.79	79.88	49.44	<.0001

**Table 3:** Estimated utilities or preference weights from TTO and VAS assessments of the HLH states

**Health state Descriptions**	**TTO Utility value**	**95% Confidence Limits**		***P*-value versus active HLH disease**
Active HLH	0.32	0.24	0.39	reference
Active HLH on chemotherapy	0.26	0.17	0.34	0.05
Active HLH with CNS involvement	0.27	0.18	0.35	0.09
Undergoing HSCT	0.18	0.07	0.28	0.00
Graft versus Host disease	0.07	−0.04	0.17	<.0001
Transplant Success	0.72	0.67	0.77	<.0001
End of life	−0.17	− 0.27	− 0.07	<.0001
**Health state Descriptions**	**VAS value**	**95% Confidence Limits**		***P*-value versus active HLH disease**
Active HLH	31	27	34	reference
Active HLH on chemotherapy	28	25	32	0.093
Active HLH with CNS involvement	27	24	31	0.009
Undergoing HSCT	23	20	26	<.0001
Graft versus Host disease	21	18	23	<.0001
Transplant Success	55	52	59	<.0001
End of life	23	20	26	<.0001

In models including age, gender, education and employment it was found that none of these variables related to the participant characteristics impacted valuations in either the TTO or the VAS analysis, which adds to the robustness of the results.

^a^GCSEs are the school exams typically taken when children are 16 years old in the UK

## Discussion

The current study estimated utility values associated with stage of disease, treatment and complications related to primary HLH in the UK. Health states were developed from a literature review and qualitative interviews with parents of children with HLH and clinicians in a series of semi-structured interviews and review. Once developed, a time trade-off methodology was employed to elicit utility values for each of the health states from a broadly representative sample of the general public in the UK.

Studies with the aim of estimating utilities in rare orphan pediatric indications are very challenging for a number of reasons. The very low prevalence of the condition makes prospective data collection, especially outside of a trial program, almost impossible. For very young children there are no validated measures of quality of life (such as EQ-5D-Y) that are suitable, as the patients are usually infants. As a fallback therefore in this study we have reverted to the vignette method for estimating utilities. The vignette method has methodological limitations partly related to the fact that it is difficult to validate the content of the descriptions. Also, there is an underlying assumption with the method that a single description of a health state can describe all patients in that state which is of course a simplification. On the positive side however, it is possible for us to include very specific information related to the disease and treatment in the vignette descriptions which means that we are not restricted to assessing HRQOL in terms of the questions in a generic instrument such as EQ-5D or the Health Utilities Index. But this itself may lead to a lack of standardization. While the vignette method has limitations, it does allow us a way of estimating utilities which incorporates the views and experiences of families affected by the disease and the treating physicians. And it provides a mechanism for estimating utilities when few other options are available.

The current study also didn’t include any parents whose children had died from primary HLH or HSCT which is an important limitation. This is a possible outcome of the disease and treatment and the study team acknowledges that the qualitative interviews were not able to capture this from a parent perspective.

The TTO and VAS values for almost all health states are low, the one exception being the cure state. These low values are consistent with the severity of the disease. It could be argued that the rarity of the disease and the nature of the impact of the disease on patients’ lives means that HLH is really beyond most people’s experience and so they are difficult for people to understand. For these reasons it may be difficult for people to reliably judge the vignettes. However, the participants in this study had no significant challenge in being able to understand the severity of the states in terms of their impact on HRQOL and maybe what they would be willing to give up to avoid these states. Without independent research it is difficult to verify whether the low scores are valid. We searched the literature in pediatric oncology to identify studies which collected data from children undergoing a bone marrow transplant (BMT). These searches suggested that most studies reported data from patients only after they have recovered [12]. Aristides et al. report values for adults with acute lymphoblastic anemia; with 0.50 for partial recovery after BMT and 0.30 for progressive disease [13]. Similarly, in acute myeloid leukemia Castejón et al. (2018) report utility estimates of 0.28 for BMT; and 0.36 for chemotherapy during a month long hospital stay [14].

The utility weights collected in the present study will support the estimation of the cost effectiveness of treatments in HLH. They were designed to support the assessment of emapalumab-lzsg; but they could be used for any treatment that affects the treatment pathway for people with HLH. The values also help to communicate the degree of burden that HLH patients face and the current extent of unmet need. Despite the limitations this is a methodological approach which can support the work of decision makers when assessing ultra-orphan treatments such as emapalumab-lzsg.

## Conclusion

In order to estimate utilities for cost-effectiveness analysis in this study we developed detailed descriptions of HLH related health states. These descriptions were developed based on information from a literature review alongside information from interviews with physicians and families affected by the disease. After rounds of review these were then assessed in TTO interviews with the general public. We believe that given the methodological challenges in assessing ultra-orphan diseases like this the results represent the best available method to generate utility values outside of a clinical trial and support future cost-effectiveness analyses. In due course it would be useful to prospectively assess HRQL burden in the disease perhaps through a disease registry, and also evaluate long-term impacts of primary HLH.

## Appendix: Health states

**Before transplant (active disease).**

- You have a life-threatening disease of the immune system which is genetic. You are waiting for a stem cell transplant (a transplant which replaces unhealthy bloody cells with healthy ones from the blood or bone marrow) which your doctor has said, if successful, would cure the condition. You are dependent on this transplant in order to survive. You are also receiving intravenous and oral medication. **You are in hospital.**
- You have had persistent, high fever for a long time which has led you to stay in hospital for weeks. You also have an enlarged spleen and have inflammation in your liver. Your doctor has told you that you have a low red blood cell count. You have anaemia (low iron in blood). You are at high risk of developing an infection. You **have** low energy and **often** experience loss of appetite**.** You are also likely to bruise very easily. You have **some pain** in your body which is being controlled by medication.
- **You are receiving treatment allowing you to become stronger in order to undergo a transplant. You have to wait until a suitable donor becomes available. You have to eat through a tube in your nose.** You can only walk around the restricted parts of the hospital due to the risk of infection. You have **some** trouble walking up and down the stairs. You are able to wash and dress yourself with some help.
- You are **not** able to go out. Your immediate family is with you.
- You may experience **some** difficulty paying attention to tasks like reading and playing games.
- You feel **anxious** and **angry** as you are spending so much time in the hospital. You also feel **upset** and **frustrated** that you are not able to do much.

**Before transplant (active disease on chemotherapy).**

- You have a life-threatening disease of the immune system which is genetic. You are waiting for a stem cell transplant (a transplant which replaces unhealthy bloody cells with healthy ones from the blood or bone marrow) which your doctor has said, if successful, would cure the condition. You are dependent on this transplant in order to survive. You are also receiving intravenous and oral medication including chemotherapy. **You are in hospital.**
- **The treatment has left you feeling very tired most of the time. You have lost your appetite. You sometimes feel nauseous and have been vomiting. You experience diarrhoea some of the time.**
- You have had persistent, high fever for a long time which has led you to stay in hospital for weeks. You also have an enlarged spleen and have inflammation in your liver. Your doctor has told you that you have a low red blood cell count. You have anaemia (low iron in blood). You are at high risk of developing an infection. You are also likely to bruise very easily. You have **some** pain in your body which is being controlled by medication.
- You are receiving treatment allowing you to become stronger in order to undergo a transplant. You have to wait until a suitable donor becomes available. You can only walk around the restricted parts of the hospital due to the risk of infection. You are **too** tired to walk up and down the stairs. You are able to wash and dress yourself with some help.
- You are **not** able to go out. Your immediate family is with you.
- You may experience **some** difficulty paying attention to tasks like reading and playing games.
- You feel **anxious** and **angry** as you are spending so much time in the hospital. You also feel **upset** and **frustrated** that you are not able to do much.

**CNS disease + HLH.**

- You have a life-threatening disease of the immune system which is genetic. You are waiting for a stem cell transplant (a transplant which replaces unhealthy bloody cells with healthy ones from the blood or bone marrow) which your doctor has said, if successful, would cure the condition. You are dependent on this transplant in order to survive. You are also receiving intravenous and oral medication. **You are in hospital.**
- **You occasionally experience fits (seizures) and some disorientation. You sometimes experience headaches. You are monitored regularly and are receiving treatment for your seizures. You may have problems with sleep. You will have to stay in hospital for a prolonged time due to treatment.**
- You have had a persistent fever for a long time which has led you to visit the hospital for weeks. You also have an enlarged spleen and are experiencing inflammation in your liver. Your doctor has told you that you have a low red blood cell count which has led to anaemia. You are at high risk of developing an infection. You have **low** energy and **often** experience loss of appetite. You are also likely to bruise very easily. You have **some** pain in your body which is being controlled by medication.
- You can only walk around the restricted parts of the hospital due to the risk of infection. You have **some** trouble walking up and down the stairs. You are able to wash and dress yourself with some help.
- You are **not** able to go out. Your immediate family is with you.
- You may experience **some** difficulty paying attention to tasks like reading and playing games.
- You feel **anxious** and **angry** as you are spending so much time in the hospital. You also feel **upset** and **frustrated** that you are not able to do much.

**Undergoing-transplant.**

- You have a life-threatening disease of the immune system which is genetic. **You need to have a stem-cell transplant (a transplant which replaces unhealthy bloody cells with healthy ones from the blood or bone marrow) and have received some treatment to prepare you for the transplant. You are hopeful but there is a chance that the transplant may not be successful in curing your condition. If it is not successful, you may require a second transplant.** You are also receiving intravenous and oral medication. **You are in hospital, confined to your room. You need daily blood transfusions and blood tests.**
- You have persistent, high fever for a long time which has led you to visit the hospital for weeks. You also have an enlarged spleen and experience inflammation in your liver. Your doctor has told you that you have a low red blood cell count. You have anaemia (low iron in blood). You are at high risk of developing an infection**.** You have **reduced** energy and **reduced** appetite. You are also likely to bruise very easily. You have some pain in your body which is being controlled by medication.
- **You are restricted to a single room due to risk of infection**. You are **not able** to walk up and down the stairs. You need help to wash and dress yourself.
- You are **not** able to go out. Your immediate family is with you.
- You may experience **some** difficulty paying attention to tasks like reading and playing games.
- You feel **very anxious** and **angry**. You feel **very upset** and **frustrated** that you are not able to do much.

**Graft vs host disease.**

- You have a life-threatening disease of the immune system which is genetic. **Some time ago, you received some treatment to prepare you for stem cell transplant (a transplant which replaces unhealthy bloody cells with healthy ones from the blood or bone marrow). You have just received a stem cell transplant which has required you to take additional treatments such as steroids**. You are also receiving intravenous and oral medication. **You are in hospital, confined to your room.**
- **You have a skin rash which is itchy and can cause discomfort. You may have a greater risk of infections. You may have severe diarrhoea which causes stomach cramps and makes it difficult to walk around. You are going to stay in hospital for longer due to this.**
- You had persistent, high fever for a long time which is coming down now. You also had an enlarged spleen and experienced inflammation in your liver. Your doctor told you that you had a low red blood cell count. You have anaemia (low iron in blood). You are at a high risk of developing an infection. You have **no** energy and **no** appetite. You **have significant** pain in your body which is being controlled by medication.
- **You are in isolation**. You are **not able** to walk up and down the stairs. You need help to wash and dress yourself.
- You are **not** able to go out. Your immediate family is with you.
- You may experience **some** difficulty paying attention to tasks like reading and playing games.
- You feel very anxious and **angry**. **You get depressed that you are not getting better.** You feel **very upset** and **frustrated** that you are not able to do much.

**Successful transplant.**

- You previously had a life-threatening disease of the immune system which is genetic. **You received a stem cell transplant (a transplant which replaces unhealthy bloody cells with healthy ones from the blood or bone marrow) which cured the condition.** You may still need intravenous treatment once a month in the hospital. **You are living back at home.**
- **You are recovering** but you are still at high risk of developing an infection. **Your energy levels and your appetite have returned. You no longer feel pain.**
- You **are careful** about going to crowded places due to risk of infection. You **don’t need** any help to wash and dress yourself. You find it **somewhat** difficult to walk up and down the stairs.
- You **don’t go out** much due to the risk of infection.
- You may experience **some** difficulty paying attention to tasks like reading and playing games.
- You **sometimes** feel **anxious** about your treatment and what you have gone through. **You sometimes feel frustrated that you still have to be careful about not getting infections.**

**End of life.**

- You have a life-threatening disease of the immune system which is genetic. **Previously, you received multiple stem cell treatments (a transplant which replaces unhealthy bloody cells with healthy ones from the blood or bone marrow) in hospital but they were unsuccessful**. **You are now receiving intravenous and oral treatment to make you feel comfortable**. **You are at home, confined to bed.**
- You have had persistent, high fever for a long time. You also have an enlarged spleen and have inflammation in your liver. Your doctor has told you that you have a low red blood cell count. You have anaemia (low iron in blood). You are at high risk of developing an infection. You have **extremely** low energy and experience loss of appetite. You are also likely to bruise very easily. You have **extreme** pain in your body.
- **You need help to wash and dress yourself**.
- You are not able to go out. Your immediate family comes to visit you.
- You experience **difficulty** paying attention to tasks and concentrating.
- You feel **very** anxious and angry. **You are worried about the future**. You feel **extremely** upset and frustrated that you are not able to do much.

## Abbreviations

AIC: Akaike’s Information Criterion
CNS: Central nervous system
GEE: Generalized estimating equations
GVHD: Graft vs host disease
HLH: Hemophagocytic lymphohistiocytosis
HRQL: Health related quality of life
HSCT: Hematopoietic stem cell transplant
HTA: Health Technology Assessment
NICE: National Institute for Health and Care Excellence
QALY: Quality adjusted life year
QIC: Quasi Information Criterion
SCT: Stem cell transplant
TTO: Time trade off
VAS: Visual analogue scale
